# Description of a Cretaceous amber fossil putatively of the tribe Coprophilini (Coleoptera, Staphylinidae, Oxytelinae)

**DOI:** 10.3897/zookeys.782.27733

**Published:** 2018-08-17

**Authors:** György Makranczy, Shûhei Yamamoto, Michael S. Engel

**Affiliations:** 1 Department of Zoology, Hungarian Natural History Museum, H-1088 Budapest, Baross utca 13, Hungary Hungarian Natural History Museum Budapest Hungary; 2 Integrative Research Center, Field Museum of Natural History, 1400 S Lake Shore Drive, Chicago, IL 60605-2496, USA Field Museum of Natural History Chicago United States of America; 3 Division of Entomology, Natural History Museum and Department of Ecology & Evolutionary Biology, University of Kansas, 1501 Crestline Drive – Suite 140, Lawrence, Kansas 66045-4415, USA University of Kansas Lawrence United States of America; 4 Division of Invertebrate Zoology, American Museum of Natural History, Central Park West at 79th Street, New York, New York 10024-5192, USA American Museum of Natural History New York United States of America

**Keywords:** Burmese amber, Cenomanian, Mesozoic, Myanmar, new genus, new species

## Abstract

An unusual and well-preserved fossil staphylinid is described and figured from a single specimen in Upper Cretaceous Burmese amber. *Gollandiaplanata***gen. et sp. n.** is tentatively placed in the extant oxyteline tribe Coprophilini, although it lacks a few characteristic features of present-day members of the group, likely indicating it to be either a stem group of the tribe or prove to be distinct pending future discoveries. The discovery of this genus suggests that early oxytelines were more morphologically diverse during the Cretaceous and their evolutionary history was more complicated than previously documented. Tribal placement as regards fossil oxyteline taxa is discussed.

## Introduction

The staphylinid subfamily Oxytelinae Fleming, 1821 is a relatively large group with over 2049 valid extant species placed in 40 genera (Thayer 2016). Oxytelines are distributed worldwide and have proven to be remarkably diverse. This diversity is so far not fully known as in several geographical areas the species are incompletely known, in many cases a vast portion (up to 80%) still await formal description. The generic revision of Herman (1970), which was also a first attempt at a phylogenetic analysis, set the foundation for a better understanding of relationships within the subfamily and for all subsequent revisionary and evolutionary research on oxytelines.

Despite Herman’s (1970) monumental study, the classification of tribes within Oxytelinae remains unsettled. The most primitive oxytelines (formerly as tribe Deleasterini, sensu Makranczy 2006, based on abdominal segments with only one pair of laterosclerites) are now often split into three separate tribes: Deleasterini Reitter, 1909, Euphaniini Reitter, 1909, and Syntomiini Böving & Craighead, 1931. The higher oxytelines, comprising 98% of the described valid species in the subfamily, are variously classified in the more primitive Coprophilini Heer, 1839, as well as the now widely accepted Blediini Ádám, 2001 and Planeustomini Jacquelin du Val, 1857. The more derived clades seem to belong into a once again unified Oxytelini (sensu Makranczy 2006), although this remains debated and awaits support from molecular studies.

The tribe Coprophilini is currently without identified synapomorphies, and is instead defined by a lack of features of the more derived lineages (i.e., it is presently circumscribed by putative plesiomorphies, may be paraphyletic, and is in need of considerable revisionary and phylogenetic exploration). The extant coprophilines are characterised by the following combination of traits: mesocoxae narrowly separated by mesosternal process or contiguous, tarsal formula 5-5-5, abdominal segments with two pairs of laterosclerites, and with only six sternites visible. According to Herman (2001), the tribe contains three extant genera, *Coprophilus* Latreille, 1829, *Coprostygnus* Sharp, 1886, and *Homalotrichus* Solier, 1849, although a poorly described genus, *Coprotrichus* Hayashi, 2005 (based on a single species from Japan) was later added and is presently considered as valid but requires detailed study. The number of species in these genera is not great; *Coprophilus* stands in the last catalogue with 30 valid species (Herman 2001), *Homalotrichus* and *Coprostygnus* are being revised by the first author, standing with at least 13 and five species, respectively (Makranczy, unpubl. data). *Coprophilus* is widespread in the Northern Temperate zone, *Homalotrichus* is known from Australia (including Tasmania) and South America, while *Coprostygnus* is confined to New Zealand. It is remarkable that all of these species prefer cold climates, often occurring at the highest elevation where one can find oxytelines, up to 4000–4200 m.

The various records of fossil Oxytelinae and its related subfamilies were summarized by Cai et al. (2017). Hitherto, the only definitive fossil Coprophilini described is a rather poorly preserved compression fossil (without counterpart), *Mesocoprophilusclavatus* Cai & Huang, 2013. A further fossil genus, *Sinoxytelus* Yue, Zhao, & Ren, 2010 was subsequently transferred to Coprophilini, but this placement is tentative as the abdomen has basolateral ridges, a trait of Oxytelini. The genus originally contained three extinct species (*Sinoxyteluseuglypheus* Yue, Zhao, & Ren, 2010, *S.breviventer* Yue, Zhao, & Ren, 2010, and *S.longisetosus* Yue, Zhao, & Ren, 2010) from the Yixian Formation (Lower Cretaceous, ca. 126 Mya, 41°36’44”N, 120°49’48”E), Liaoning, China (Yue et al. 2010), and a fourth taxon, *S.transbaicalicus* Cai, Yan, & Vasilenko, 2013 was later described from the Urey beds (Lower Cretaceous, 50°38.730’N, 112°50.338’E), Transbaikalia, Russia (Cai et al. 2013). Here we describe a new genus and species from the Upper Cretaceous amber of northern Myanmar.

This new taxon represents the second fossil occurrence of Oxytelinae documented from Mesozoic amber, and is also the oldest amber inclusion presently recorded for the subfamily along with *Prajnatianmiaoae* Lü et al., 2017, a species of Thinobiini (a more derived tribe) described from the same deposit.

## Materials and methods

Specimen photography was done with the amber piece mounted on a small plastic plate surface with a drop of viscous glycerine covered with a glass cover slip to eliminate distortions from the otherwise rounded amber surface. The habitus photographs were made with a Canon 5D Mark III camera and C Canon MP-E 65mm f/2.8 1–5× macro lens with two Canon Macro Twin Lite MT-24EX flash units and a Canon Speedlite 430EX III-RT flash unit standing on the shoe foot directly in front of the specimen (shooting directly into the head of the specimen). The light was diffused by a single ring of mylar. Raw photograph files were imported to Adobe Lightroom 5.7.1, and layers stacked with ZereneStacker (Richland, Washington, USA). Details of the specimen were photographed with a Canon EOS 6D camera attached to a Leica M205 C stereomicroscope with the help of a Canon EOS Utility 3.4.30.0 software, before being stacked using the same software as previously mentioned. Abbreviations for measurements are defined as follows:

**HW** head width with compound eyes;

**TW** head width at temples;

**PW** maximum width of pronotum;

**SW** approximate width of shoulders;

**MW** maximum width of elytra;

**AW** maximum width of abdomen;

**HL** head length at the middle-line from front margin of clypeus to the beginning of neck;

**EL** compound eye length;

**TL** length of temple;

**PL** length of pronotum at the middle-line;

**SL** length of elytra from shoulder;

**SC** length of elytra from hind apex of mesoscutellum;

**FB** fore-body length (combined length of head, pronotum, and elytra);

**BL** approximate body length.

The specimen is exceptionally well preserved but in a rather unfortunate position within the amber piece. Under UV-light examination it can be seen that the specimen is sitting within a dip in the internal flow of the amber (the amber flowed in layers when it was originally exuded from the tree and the beetle is in a dip within these flows). The result of its placement within the dip in the flow means ideal, clear images cannot be produced at high magnification unless some of the flows can be polished away. Whoever made the original preparation (probably local workers in Myanmar) polished the specimen too close to the amber surface and cut the piece poorly. The result is that it is now impossible to cut and polish the piece closer to minimize the optical impact of the flow lines.

The recent commercial amber mines are located in the Hukawng Valley (26°16.5’N, 96°34.0’E), Kachin, northern Myanmar. The minimum age of the amber is estimated to be 98.79 ± 0.62 Mya (by radioisotope dating of zircon crystals obtained from the volcanoclastic matrix, Shi et al. 2012), and so just slightly into the base of the Cenomanian. Cruickshank and Ko (2003), Ross et al. (2010), and Grimaldi and Ross (2017) provide a geological account of the deposits, and these authors also note that many of the amber pieces appear to have at some point eroded from sediments and been redeposited, at least suggesting the possibility that the inclusions could be slightly older, perhaps from the latest Albian. Recent studies speculate that the West Burma Plate/Block (west of the Sagaing fault line in Myanmar) was of Gondwanan origin (Poinar 2018), and the resin-producing tree was hypothesized to be of *Agathis* Salisb. (Araucariaceae) (Poinar et al. 2007). The paleoclimate of Burmese amber producing forests were suggested to be tropical with an average temperature range of 32–55 °C (Grimaldi et al. 2002; Ross et al. 2010).

## Systematic Palaeontology

### Family Staphylinidae Latreille, 1802

#### Subfamily Oxytelinae Fleming, 1821

##### Tribe Coprophilini Heer, 1839

###### Gollandia gen. n.

Taxon classificationAnimaliaColeopteraStaphylinidae

http://zoobank.org/910F415D-CEA9-4E31-BF75-801F8A73BE80

####### Type species.

*Gollandiaplanata* sp. n., (described below).

####### Diagnosis.

*Head*. Head somewhat retracted under large pronotum; head capsule rather short. Epistomal sulcus not well visible, but presence suggested by a tranvserse ‘run’ of air between amber and cuticle. Supraantennal prominences weak. Antennomeres with long tactile setae near apices (prominent on articles 3–11). Labial palp trimerous, basal two palpomeres rather stout, last palpomere thin. Labrum with two thick, forward-directed setae. Mandibles not prominent, apices acute. Maxillary palp tetramerous, basal three palpomeres moderately elongate, last palpomere much wider and long, not reduced, apex pointed. Gular sulci seemingly widely separated at base but confluent anteriorly (this area is not well visible as preserved). Neck separated by gentle constriction and (at least laterally) a groove. *Thorax*. Pronotum strongly explanate, margin slightly reflexed, marginal bead present, lateral edge finely serrate/sinuous. Laterally with a strong seta at each of ‘anteroangularis’ and ‘lateralis’ positions, plus strong seta on both sides well inside lateral margin at about 1/3 length, posterior edge appearing slightly concave (might be artefact of preservation). Pronotal disc with shallow impressions, with fine and dense punctation and setation. Procoxae contiguous, projecting; procoxal fissure present and open (Figure 7). Mesoscutellum (Figure 8) with apex exposed and somewhat impressed without distinct pattern. Elytra finely and randomly punctate. Mesocoxae narrowly separated by mesosternal process (Figure 9). Legs slender (metatibia especially elongate), with regular rows of tibial spines (more slender than strong), and a conspicuous mesotibial spur (and a second spur half size at half-length towards femoral joint). Tarsal formula 4-4-4, no tarsal lobes (Figure 10), but empodial setae strong (Figure 11). Elytra with epipleural ridge, seemingly with a fine and shallow dorsal groove following it from inside, epipleura strongly deflexed and rather wide but epipleural fold thin to inconspicuous. Post-scutellar area with a pair of elongate impressions along suture. Shoulders prominently developed, narrowly rounded, even slightly projecting forward in relation to anterior edge at mesoscutellar area, posterior margin slightly oblique but straight from suture to outer 3/4, slightly incurved (concave) in outer 1/4 thereby producing a somewhat sharp outer corner in dorsal view. *Abdomen*. Abdomen with only six visible segments (not counting segments IX–X, often retracted under VIII), second abdominal segment not developed. With two pairs of laterosclerites. Apex of tergite VII seemingly without well-developed palisade fringe (difficult to judge; an air bubble under this structure obscures almost its entire width), apex of segment conspicuously widening (not narrowing to base of next segment), surface somewhat concave. Tergite VIII with apical edge truncate medially or slightly concave. Apex of sternite VIII without modification.

####### Differential diagnosis.

All extant Coprophilini have a 5-5-5 tarsal formula, and even the fossil genus *Mesocoprophilus* has five tarsomeres, so the 4-4-4 condition in *Gollandia* is significant. The new genus differs greatly from *Mesocoprophilus* in the antennal structure, stout and short in *Mesocoprophilus*, slender and elongate with well-developed tactile setae on all antennomeres in the present fossil. The neck (lateral constriction, postoccipital groove) also differentiates this genus from *Mesocoprophilus* where these features are absent. The lack of striae or puncture rows on the elytra makes this genus distinct from all extant Coprophilini, while a distinction from *Mesocoprophilus* cannot be made as that fossil lacks its dorsal portion (Cai and Huang 2013). The present fossil is also peculiar in the slender and elongate appendages. The present-day representatives of Coprophilini lack such strongly formed, almost forward-projecting shoulders and the new genus has more slender antennae and palp, more slender tibiae, a procoxal cavity far removed from the pronotal margin, a prominently explanate pronotum, and the mesosternal process extending much more posteriorly. Two unusual traits for this subfamily are the posteriorly slightly incised elytral corners and the cylindrical, wide apex of segment VII (not narrowing to the base of segment VIII), both features otherwise characteristic of the subfamily Aleocharinae.

####### Systematic placement.

The only feature that clearly unites the fossil with extant Coprophilini is the lack of the well-developed second sternite. Beyond that, the head shape is reminiscent of *Homalotrichus*, while the pronotum bears some similarity to that of some *Coprophilus* (e.g., *Coprophilusstriatulus* (Fabricius, 1793) plus its close relatives) and to a lesser extent some *Homalotrichus* (e.g., *Homalotrichusimpressicollis* Solier, 1849), but none of these are as explanate as in the fossil.

####### Etymology.

The new genus is named after Susan Golland, exhibition developer at the Field Museum of Natural History, Chicago, whom the first author met at 10:32am on 14 March 2018 in front of Crystal Maier’s office. The fossil specimen described here was shown to him by the second author later on the same day. The gender of the name is considered feminine.

###### Gollandia planata sp. n.

Taxon classificationAnimaliaColeopteraStaphylinidae

http://zoobank.org/CC08CCA8-D767-4E16-BB55-5D343BE06492

Figures 1–2 , 3–4 , 5–6 , 7–11

####### Holotype.

Sex unknown, probably male, in a flattened drop shaped, light yellow amber piece (20.0 × 9.9 × 4.5 mm, 0.98g): “FMNHINS 3729858 ex S. Yamamoto collection (SYAC0482)” deposited in Field Museum of Natural History (Chicago, USA).

####### Locality and horizon.

Noije Bum hill near Tanai Village, SW part of Hukawng Valley (SW of Maingkhwan), Kachin State, northern Myanmar; lowermost Cenomanian, Upper Cretaceous.

####### Diagnosis.

As for the genus (vide supra).

####### Description.

**Figures 1–2.**
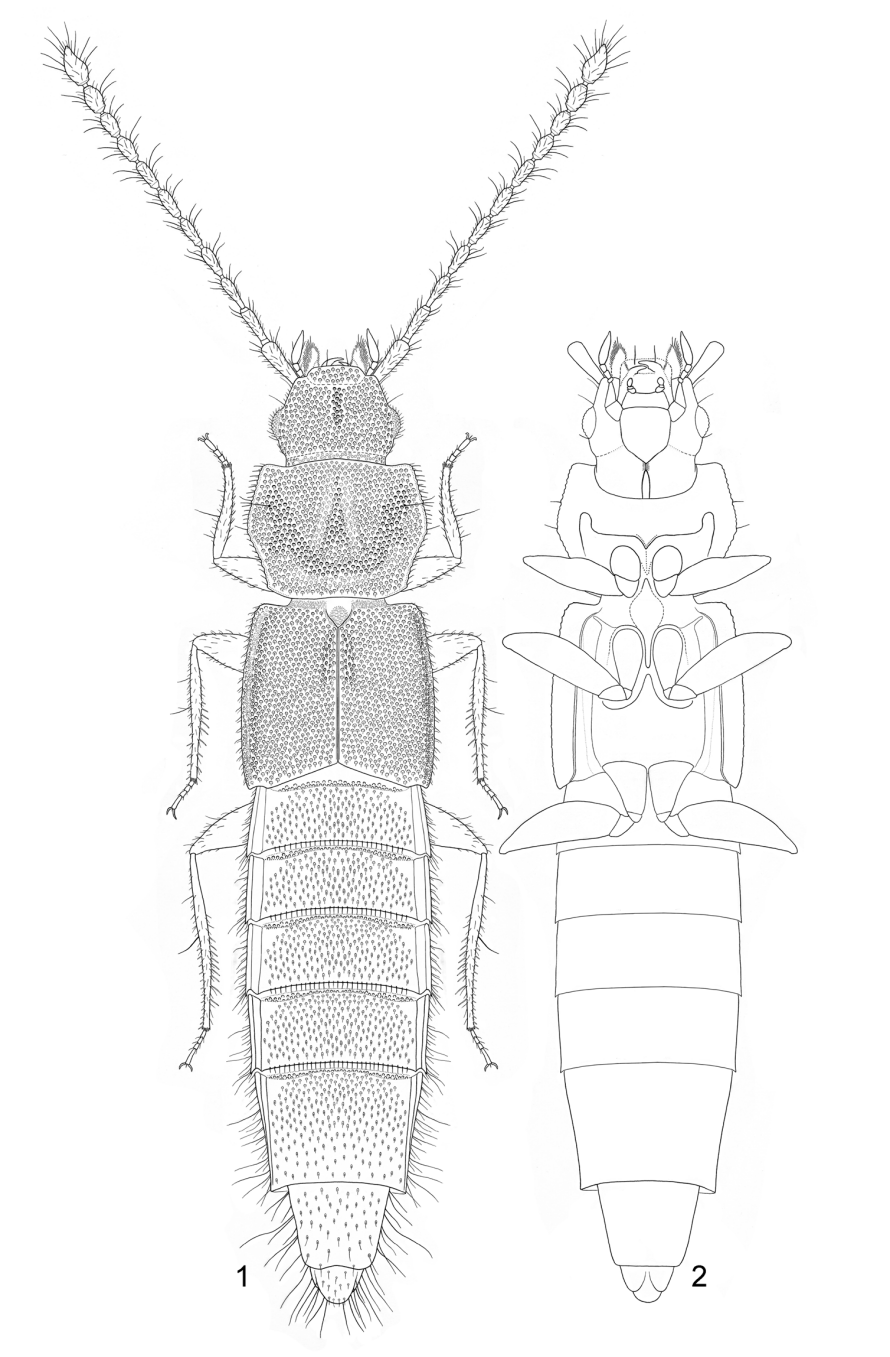
*Gollandiaplanata* gen. et sp. n. **1** habitus, dorsal view **2** sketch of main body parts, ventral view.

**Figures 3–4.**
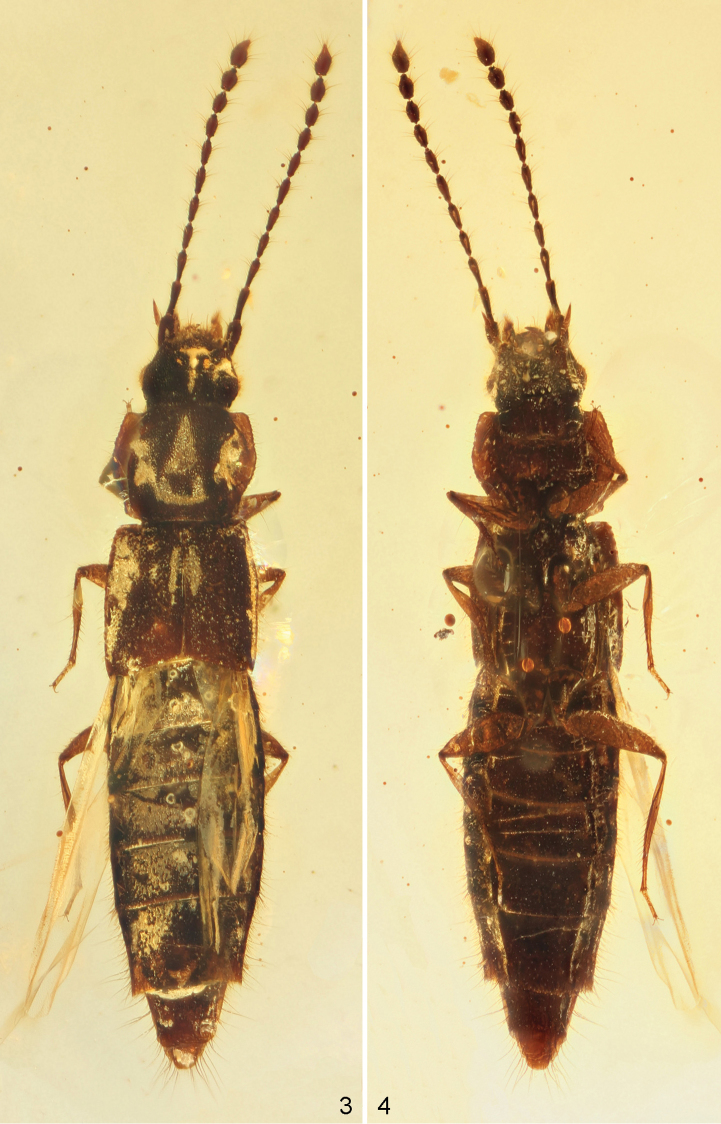
*Gollandiaplanata* gen. et sp. n. photographed with macro lens and three flash units. **3** dorsal view **4** ventral view.

**Figures 5–6.**
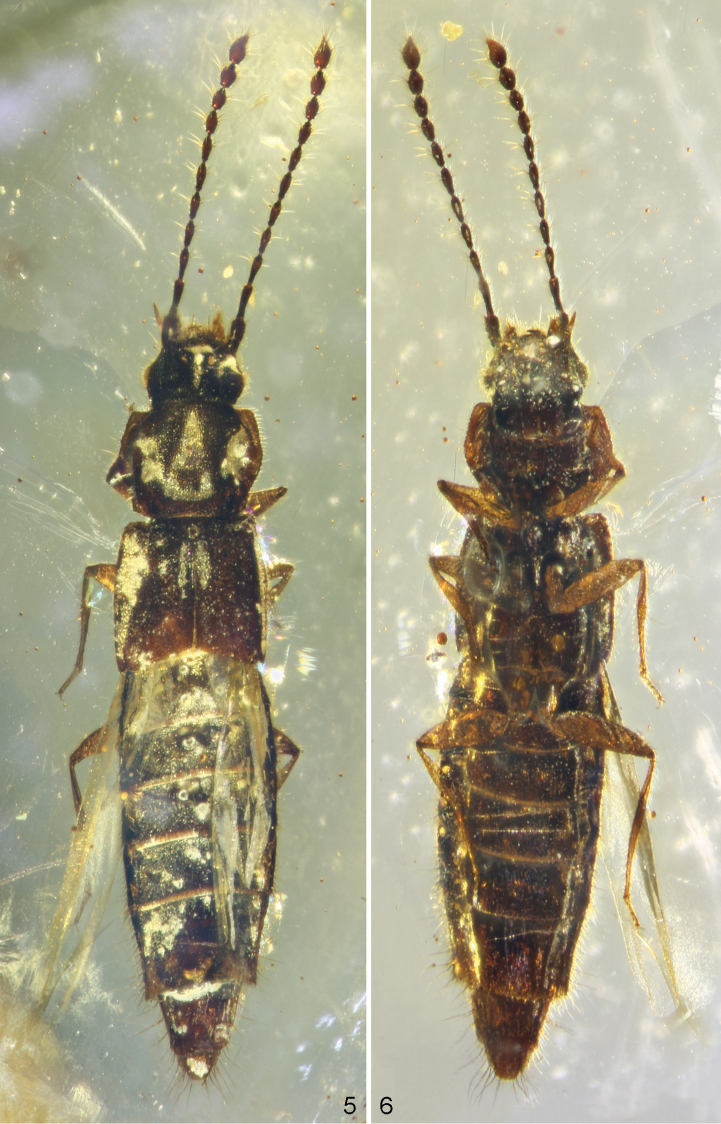
*Gollandiaplanata* gen. et sp. n. photographed through microscope, strong backlighting. **5** dorsal view **6** ventral view.

*Measurements*: HW = 0.45; TW = 0.41; PW = 0.64; SW = 0.59; MW = 0.68; AW = 0.70; HL = 0.29; EL = 0.10; TL = 0.04; PL = 0.50; SL = 0.66; SC = 0.55; FB = 1.47; BL = 3.29 mm (all measured from dorsal view). *Habitus*: General habitus as in figures 1–6. Colour reddish dark brown. Body moderately lustrous, covered with fine microsculpture and forebody finely, not very densely setose. Abdomen with longer and stronger lateral setae posteriorly. *Head*. Head rather short. Antennae rather elongate, scape almost twice as wide as pedicel and not much longer, second antennomere (pedicel) more than 3.5× as long as wide, third antennomere (first flagellomere) slender at base and almost as long as previous. Further antennomeres spindle-shaped and each with rudimentary basal dish, gently constricted above them. Antennomeres 4–7 at least 2.5× as long as wide, from antennomere 8 becoming wider, gently clubbed, last three antennomeres only about 1.5× as long as wide. Compound eyes more than 2× as long as weakly formed temples. Neck not constricting strongly. *Thorax*. Pronotum rather large, widest point slightly before middle with both anterior and posterior corners rather narrowly rounded, lateral margin slightly concave before quite acute posterior angles. Surface finely microsculptured, thereby punctation partly obscured. Disc transversally impressed before base (in a curved fashion), also with a semi-triangular mid-longitudinal impression anteriad; rather large but shallow paralateral depressions on sides. *Elytra*. Elytra together just slightly broader than pronotum, trapezoidal, shoulders well developed, narrowly rounded, even slightly projecting forward in relation to anterior edge at mesoscutellar area. Dorsal surface finely punctate and setose, no major lateral setae, epipleural ridge with moderately long setae at regular intervals. *Abdomen*. Sides of abdomen gently curved, almost parallel. Surface of tergites with moderately fine, longitudinally elongate punctures, apical edges of tergites (up to tergite VI) with row of equal-sized setae at regular intervals. Specimen is without any feature suggesting strong sexual dimorphism. No genital traits observable.

####### Etymology.

The specific epithet is a Greek adjective derived from *platys* (= wide) and refers to the pronotum of the species being unusually explanate.

####### Preservation.

The specimen is exceedingly well preserved, with the hind wings unfolded over part of the abdomen, minor air bubbles under segmental edges, a thin air layer over some sculptured dorsal parts, and the ventral side exceptionally clearly visible. As explained before, the specimen is sitting within a layer of resin covered by another layer, and this creates an effect similar to the specimen being glued to a glass, evident in the photos of the ventral side. The legs are somewhat distorted (but each pair is almost perfect on one side). Primitive oxytelines often have distinctive coxites and styli in females (if not exposed, then setation gives them away), and in their absence the specimen is presumed to be a male.

**Figures 7–11.**
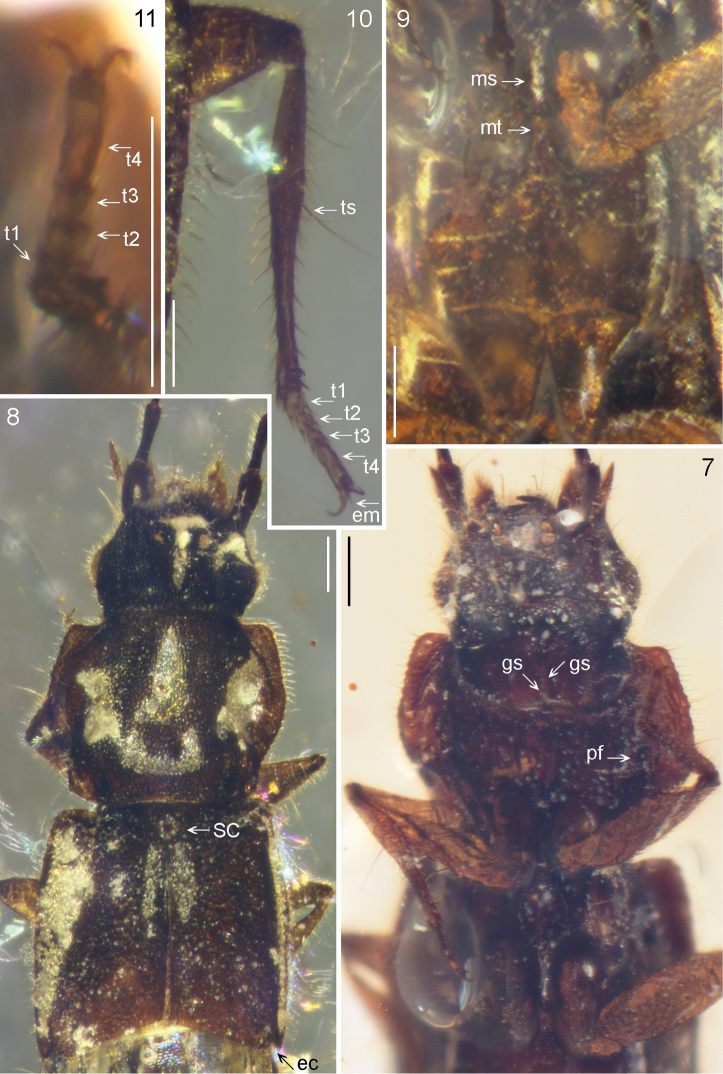
*Gollandiaplanata* gen. et sp. n. **7** head, pro- and mesothorax, ventral view **8** fore-body, dorsal view **9** meso- and metacoxae, ventral view **10** mid leg, dorsal view **11** protarsus, ventral view. t 1–4 = tarsomeres, em = empodial seta, ts = mesotibial spur, sc = mesoscutellar impression, ec = elytral posterior corner, gs = gular sulcus, pf = procoxal fissure, ms = mesosternal, and mt = metasternal process. Scale bars: 0.15 mm.

## Discussion

There can be a great debate on how to place fossil species in higher taxa when the classification is otherwise based entirely on present-day species. Naturally, fossil species placed cladistically within the crown group are not difficult, but when potential stem groups are discovered some difficulties and strong differences of opinion arise. Does one include them within the formal group, necessitating a new circumscription of the taxon boundaries in order to accommodate the fossil, or, at another extreme, more radically establish a new group for the fossil as a potential sister to its extant relatives? The latter will inevitably lead to a proliferation of meaningless and often monobasic groups based on limited characters (e.g., often lacking critical data on genital traits) and collectively forming a pectinate stem to any lineage circumscribed solely on the basis of its extant constituents, the end result becoming a classification of little explanatory power. Ideally, there one desires a balance between maintenance of a good diagnostic power and keeping the classification simple while simultaneously reflecting the hierarchical relationships supported among the various taxa being classified and granting the system maximal explanatory and predictive significance. These goals are sometimes simultaneously achieved, but more often than not the former objective becomes muddied while attempting to adequately reflect the latter. This challenge is particularly great for ancient groups, such as the Oxytelinae whose history goes back at least 150 Mya, where there has been inevitably considerable extinction over the intervening epochs between the first divergence of a given clade and its modern fauna. Cretaceous deposits will undoubtedly supply for generations to come a continuous stream of unusual fossil species that will force us to rethink our estimates of relationship and concomitantly the classifications from which they are built. Descriptive science (Grimaldi and Engel 2007) has undergone an unprecedented boom in the past few years with new techniques (tomography, confocal microscopy) providing details that bring the examination of fossil morphology more comparable to that of extant species. The result of this increase in available character data and extinct taxa will be finer phylogenetic placement of peculiar fossil species and, hopefully, greater clarity on how best to tackle each classificatory alteration as they arise.

In the case of the presently known fossil oxytelines or putative oxytelines this challenge is acute as there are limited character data available. The four fossil species presently placed in *Sinoxytelus* possess a mixture of ancient and relatively modern traits. The basolateral ridges on the abdominal tergites are a trait of the tribe Oxytelini, while the somewhat reduced second abdominal sternite suggests placement in more primitive tribes. A solid age estimate is available for part (Lujiatun Bed) of the Yixian Formation and at approximately 126 Mya, or Aptian (Chang et al. 2017). If *Sinoxytelus* is truly either a stem group to Oxytelini or Oxytelini + other higher oxytelines, then the subfamily Oxytelinae as whole was apparently already diverse by the latter part of the Early Cretaceous. It seems apparent that there has been considerable extinction within the subfamily, resulting in only a few lineages surviving and diversifying to their present state. These fossils may be rather distantly related to the ancestors of the present Coprophilini or Oxytelini, with potentially numerous additional extinct stem groups (yet to be discovered) present before the divergence of the crown groups of these latter tribes. Characters that Cai et al. (2013) cite for placing *Sinoxytelus* within Coprophilini (e.g., metasternal process slightly protruding but not meeting mesosternal process) are more indicative of particular genera rather than the tribe, as there is considerable variation within the present day genera for these traits and are not strictly diagnostic for coprophilines (Herman 1970).

The current Burmese amber fossil species has a similarly conflicting combination of traits, on the one hand it can be placed in Coprophilini because of the plesiomorphic condition of the basal abdominal segments (although the tribe is presently defined on putative plesiomorphies and so this feature alone indicates nothing more than the potential for the fossil to belong therein or to belong to stem-group coprophilines, assuming the tribe is truly monophyletic). On the other hand, the apomorphically reduced number of tarsomeres, is a derived feature, but currently cannot be considered anything more than autapomorphic for the genus. Thus, given the lack of abundant evidence definitively demonstrating its phylogenetic placement relative to modern oxyteline genera, we prefer to tentatively consider the genus as a member (a stem group member) of Coprophilini. Hopefully, in time further material and other fossil species will be discovered that will ultimately permit a clarification of its relationships along with its broader implications toward affinities among lineages of Oxytelinae, and at which time a redefinition of oxyteline tribes may be attempted.

## Supplementary Material

XML Treatment for Gollandia

XML Treatment for Gollandia planata
